# Challenges and Implications of Routine Depression Screening for Depression in Chronic Disease and Multimorbidity: A Cross Sectional Study

**DOI:** 10.1371/journal.pone.0074610

**Published:** 2013-09-13

**Authors:** Bhautesh Dinesh Jani, David Purves, Sarah Barry, Jonathan Cavanagh, Gary McLean, Frances S. Mair

**Affiliations:** 1 General Practice and Primary Care, Institute of Health and Wellbeing, University of Glasgow, Glasgow, Scotland, United Kingdom; 2 Robertson Centre for Biostatistics, Institute of Health and Wellbeing, University of Glasgow, Glasgow, Scotland, United Kingdom; 3 Mental Health and Wellbeing, Institute of Health and Wellbeing, University of Glasgow, Glasgow, Scotland, United Kingdom; Cardiff University, United Kingdom

## Abstract

**Background:**

Depression screening in chronic disease is advocated but its impact on routine practice is uncertain. We examine the effects of a programme of incentivised depression screening in chronic disease within a UK primary care setting.

**Methods and Findings:**

Cross sectional analysis of anonymised, routinely collected data (2008-9) from family practices in Scotland serving a population of circa 1.8 million. Primary care registered patients with at least one of three chronic diseases, coronary heart disease, diabetes and stroke, underwent incentivised depression screening using the Hospital Anxiety and Depression Score (HADS).

125143 patients were identified with at least one chronic disease. 10670 (8.5%) were under treatment for depression and exempt from screening. Of remaining, HADS were recorded for 35537 (31.1%) patients. 7080 (19.9% of screened) had raised HADS (≥8); majority had indications of mild depression with HADS between 8 and 10. Over 6 months, 572 (8%) of those with raised HADS (≥8) were initiated on antidepressants, while 696 (2.4%) patients with normal HADS (<8) were also initiated on antidepressants (relative risk of antidepressant initiation with raised HADS 3.3 (CI 2.97-3.67), p value <0.0001). Of those with multimorbidity who were screened, 24.3% had raised HADS (≥8). A raised HADS was more likely in females, socioeconomically deprived, multimorbid or younger (18-44) individuals. Females and 45-64 years old were more likely to receive antidepressants.

**Limitations:**

retrospective study of routinely collected data.

**Conclusions:**

Despite incentivisation, only a minority of patients underwent depression screening, suggesting that systematic depression screening in chronic disease can be difficult to achieve in routine practice. Targeting those at greatest risk such as the multimorbid or using simpler screening methods may be more effective. Raised HADS was associated with higher number of new antidepressant prescriptions which has significant resource implications. The clinical benefits of such screening remain uncertain and merits investigation.

## Introduction

Depression is up to two to three times more common in patients with chronic disease as compared to the general population [1-3]. Prevalence estimates of depression in patients with chronic diseases such as coronary heart disease (CHD), diabetes and previous stroke range from 15-25% [4–6], depending on screening methods. Depression comorbid with chronic disease is known to have detrimental effects on mortality, clinical outcomes, treatment adherence and functional outcomes such as ability to carry out activities of daily living [5,7,8]. Routine depression screening for patients with chronic disease is now being advocated [9,10]. In the UK, family practices have been incentivised to carry out annual depression screening in those with chronic disease as part of a pay for performance contract since 2006 [11]. However, the evidence supporting the clinical effectiveness of routine depression screening in the general population is equivocal [12], with recent systematic reviews arguing both in favour and against it [13,14]. There is also evidence to suggest that routine depression screening in patients with cardiovascular disease may not lead to improvement in clinical outcomes [15,16].

A range of self-report questionnaires are currently used for depression screening in primary care; however not all were designed to be used in patients with chronic disease and the best method for screening for depression in chronic disease remains uncertain. A recent meta-analysis concluded that none of the currently available depression screening questionnaires were sufficiently accurate to be recommended as a definitive tool [17]. In the UK, the National Institute for Health and Care Excellence (NICE) recommends depression screening or case finding in patients with chronic disease [18], although there is still some controversy about the benefits of such an approach [19].

It is noteworthy that most of the evidence regarding the accuracy and clinical effectiveness of depression screening in patients with chronic disease has come from randomized controlled trials with little known about the feasibility or effects of widespread screening in routine practice.

In this paper we examine the reach and effects of a programme of incentivised depression screening in chronic disease within a UK primary care setting. Our specific research questions are: (1) What is the uptake of screening for depression in chronic disease in routine primary care(2)? What is the impact of the screening questionnaire score (in this study, HADS) on the rate of antidepressants prescribed(3)? What are the demographic factors influencing a positive result on depression screening and subsequent initiation of antidepressants?

## Methods

### Ethics Statement

We received approval from the West of Scotland research ethics committee to undertake this work. Permission to analyse the anonymised data for the year 2008-9 was provided by the NHS Greater Glasgow and Clyde Enhanced Services data group, which was the authorised “guardian” of this data set.

The work involved analysis of a large routinely collected dataset. The data received was completely anonymised and the research team did not have access to patient identifiers.

In view of this, we did not seek individual patient consent to undertake this work. This was in agreement with the guidance given by the local ethics committee and the guardians of the dataset.

### Data Collection

The data reported in this paper came from the West of Scotland, with a population of circa 1.8 million served by two different health boards. The local health boards oversee a programme of incentivised depression screening in chronic disease as part of a wider chronic disease management programme of ‘Local Enhanced Services’ (LES). The Quality & Outcomes Framework (QOF) is part of the UK wide, pay for performance, General Medical Services contract for family practitioners [20]. The QOF is an incentive scheme containing a group of clinical indicators, where primary care practices are paid based on how well they score for each indicator, which is dependent on their clinical performance against any given indicator. LES are contractual arrangements at a local health board level with family practices designed to augment the basic QOF specification by incentivising additional indicators that are deemed to be particularly important by a given area and there are no penalties for non-adherence. In the areas under investigation in our study, family practices were paid under the LES scheme to carry out a comprehensive annual health assessment for patients with three common chronic diseases, CHD, diabetes and stroke, including depression screening. Besides depression screening, the annual health assessment also included assessment and management of other health related behaviours such as smoking status, alcohol consumption, diet and activity levels. The annual health assessment was usually carried out by a practice nurse and lasted for one hour. The remuneration offered varied according to disease area with £31 each for patients with diabetes, £26 each for patients with previous stroke and £23 each for patients with CHD. The remuneration was dependent on the level of coverage of indicators achieved in the health assessment, with full payment offered for >90% coverage, 3/4 payment for 75-90% coverage and half payment for 60-75% coverage.

In 2008-9, the period on which the current study is based, the comprehensive annual health assessment was offered to all patients on the practice register with one of the three aforementioned chronic diseases and was usually carried out by practice nurses. The annual health assessment protocol was based on a universal template issued by the health board and it was specific for each of the three diseases (see appendix S1). The results of the assessment were entered into the template with “Read codes” assigned to each data entry. Read codes are the coded thesaurus of clinical terms, by which clinicians in the UK record patient findings and procedures [21]. The assessment included detailed history taking, various physical examinations and blood tests, and recording of certain drugs prescribed including antidepressants, anti-psychotics and cardiovascular drugs.

Importantly, the health assessment included screening patients for depression using the depressive subscale of the Hospital Anxiety and Depression Score (HADS-D) [22]. Patients, who were noted to be under treatment for depression, were exempt from depression screening. The template recommended that patients with HADS of 11 or more should be considered for cognitive behavioural therapy or pharmacotherapy, either by the practice nurse doing the assessment or via subsequent review by the family practitioner, whichever was deemed appropriate by the practice. Patients with HADS of 8-10 were to be advised about low intensity psychosocial interventions such as self-help services or useful online resources [23,24].

### Data Processing and Analysis

The data consisted of Read codes, which identified specific measures, the value of each measure taken and the date recorded. Read code identification was based on a list provided by the health board (see appendix S1). Read code entries were converted into clinical values for analysis and independently validated by a second researcher. The validation process continued until both researchers were in agreement.

We restricted our analysis to adults aged from 18 to 90 and health assessments recorded between 01/04/2008 to 31/03/2009. We analyzed the prescription records of those patients, who did not have depression screening results recorded, for an antidepressant prescription in the 12 month observation period. We were not able to explicitly differentiate which of the patients in this category were newly prescribed antidepressants. However based on our situational knowledge of primary care practice in Scotland, that the average prescription duration is not usually longer than 90 days, we further classified these patients into those ‘likely’ to be newly started on antidepressants. If patients in this category were first prescribed antidepressants more than 90 days after the start of the observation period, they were labelled as ‘likely’ to be newly started on antidepressants without undergoing depression screening. Patients were labelled as ‘under treatment’ for depression and exempt from depression screening if: (a) they were noted to be on antidepressants based on their prescription record with no record of depression screening during the 12 month observation period (01/04/2008 to 31/03/2009) (b) the first prescription for antidepressant was issued in the first 3 months of observation period. The full list of antidepressants prescribed can be accessed from appendix S2.

The depressive subscale of HADS (HADS-D), which gives a total score of 21, was used as a screening tool. A score of ≥11 is considered a clinically significant disorder, whereas a score between 8 and 10 suggests a mild disorder [22]. We used the threshold of HADS ≥8 as a cut-off for a ‘positive screening result’ as there is evidence to suggest that this offers an optimal balance of sensitivity and specificity [25-27] and such an approach has been endorsed by national guidelines [28]. All patients who underwent depression screening were checked for a new prescription of antidepressants in the six months after the date of assessment. No reliable information was available on the number of patients who were referred for psychological therapies following their depression screening. We also did not have information on history of previous episodes of depression or history of antidepressants prescribed in the past, prior to the observation period. We considered the effects of screening for each of the diseases individually and in combination. The socioeconomic status was provided to us in the form of the Scottish Index of Multiple Deprivations (SIMD) score, which identifies small area concentration of multiple deprivations across all of Scotland in a consistent way. The SIMD score was divided into quintiles from 1-5 with Q1 representing the most deprived area [29]. Smoking status was divided into current non-smokers and smokers; alcohol status was classified into moderate (< 21 units men, <14 units women), hazardous (21-50 units men, 14-35 units women) and harmful (>50 units men, >35 units women) based on their weekly units consumption. This classification was adapted from the latest report of the Scottish Health Survey [30]. The other variables for health related behaviour such as diet and physical activity levels all had a large number of missing values and hence were excluded from all analyses.

### Statistical Analysis

The incidence of a positive screening was calculated for single diagnoses and for both 2 and 3 co-morbidities. The incidence rate was calculated as the number of positive results found as a result of depression screening (defined as HADS ≥8) of the total number of patients screened in each diagnostic or demographic subgroup. Similarly, the rate of new antidepressant treatment was calculated by dividing the number of subjects with a raised HADS who started treatment, by the total number with a raised HADS. Logistic regression models were used to examine whether age, gender, deprivation quintile and multimorbidity were associated with the odds of screened patients having a raised HADS; and on the odds of starting a new antidepressant treatment given a raised HADS following screening. Results of the regression models are presented as the odds ratios associated with either a fixed increase in the value of the predictor (for continuous predictors) or compared to the stated reference group (for categorical predictors), with 95% confidence intervals (CI) and p-values. We visualise the logistic regression analysis by presenting the corresponding predicted probabilities of new treatment depending on a patient’s age, gender and HADS, and adjusted for deprivation and comorbidity, to allow inference for other patients meeting these criteria.

We present a Kaplan-Meier plot to illustrate the time from start of monitoring to starting new treatment dependent on whether patients were screened for depression or not. All subjects were followed up for 6 months and patients who did not start treatment were censored at 6 months. Patients in the screened population were stratified by whether they had a HADS<8, 8 to 10 or ≥11.

Analysis was carried out using the R statistical software, version 15.1 [31]

## Results

### Section 1. Uptake of Depression Screening in Routine Practice

**Figure 1:**
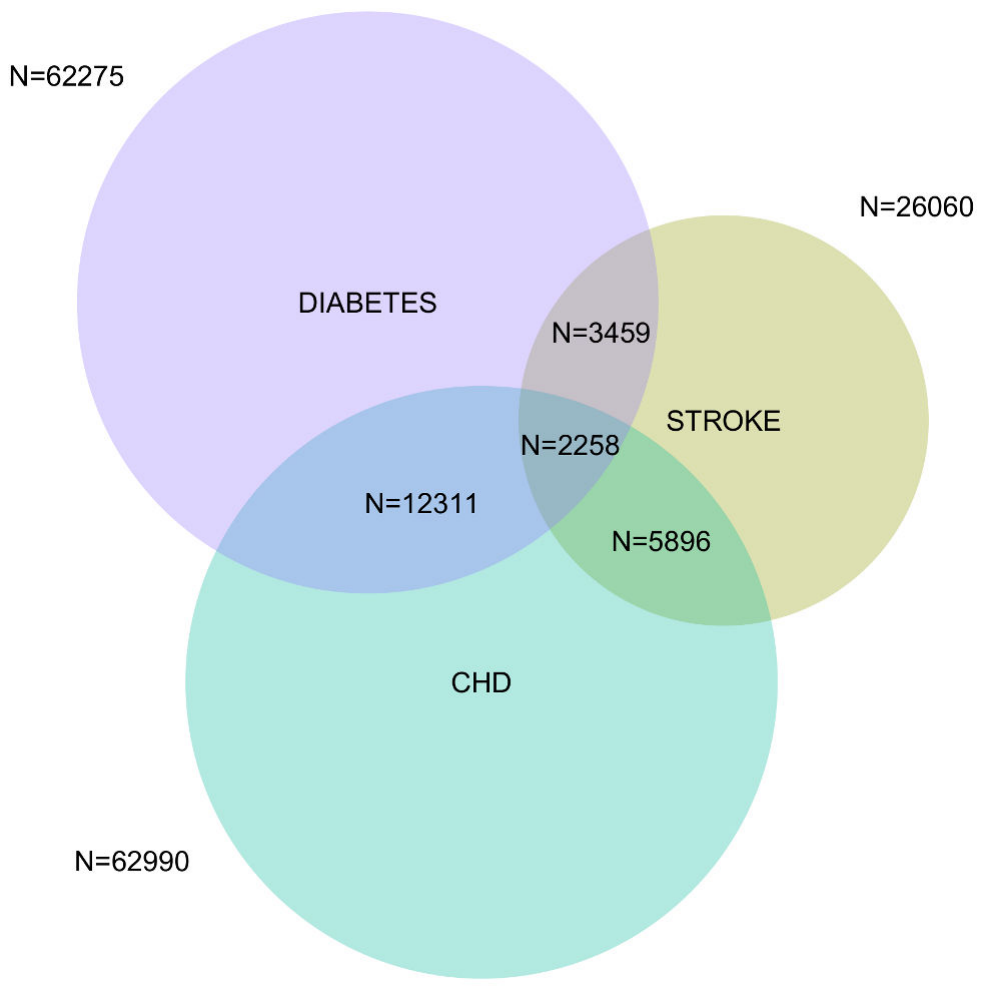
Distribution of patients across three diseases for the year 2008-09.

A total of 125,143 patients were listed as having CHD, diabetes or stroke in the year 2008-09. Figure 1 shows the distribution of patients across the three diseases and their respective proportional combinations.

**Figure 2:**
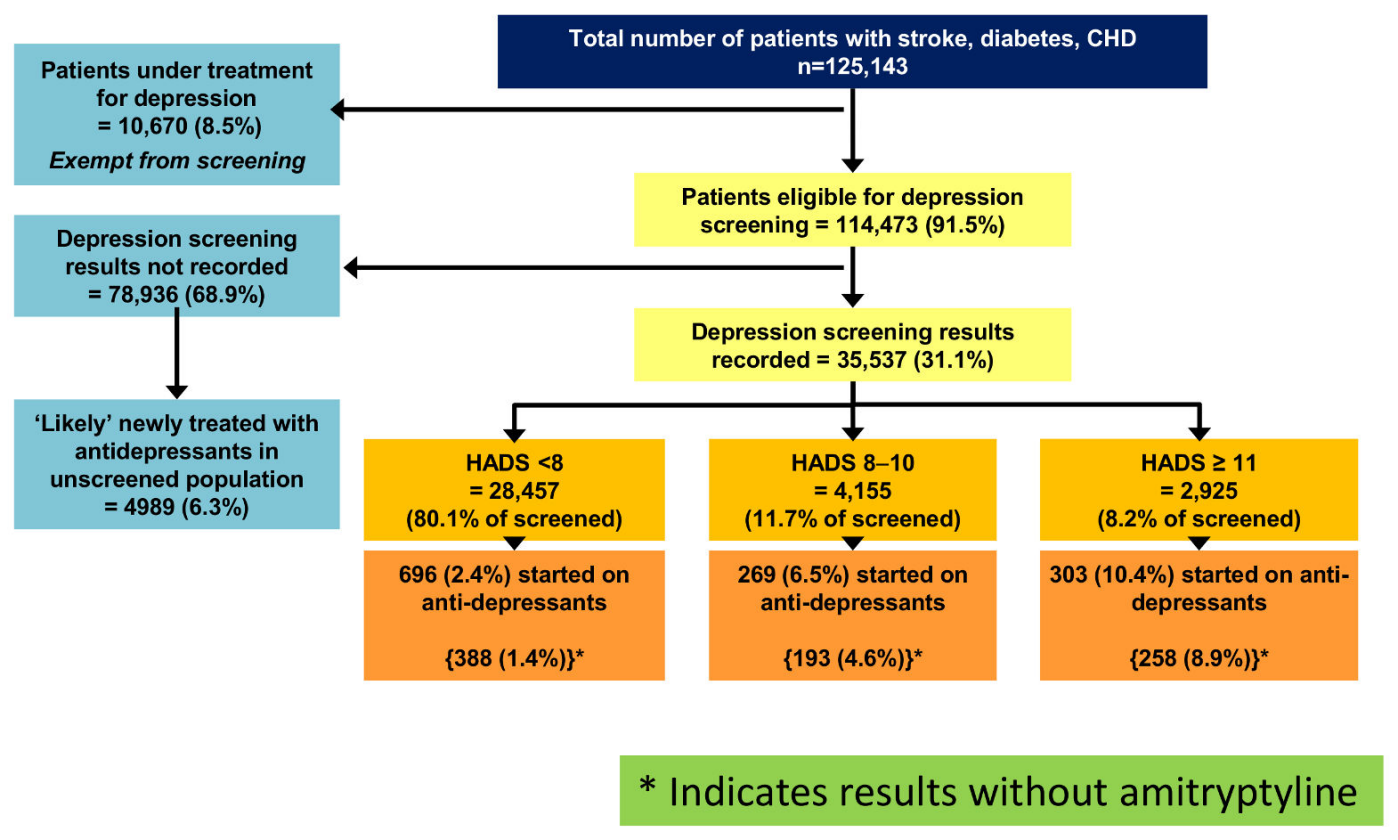
Flow chart showing the outcome of depression screening.

Figure 2 shows the distribution of patients according to their depression status. Of the total sample, 10,670 (8.5%) patients were under treatment for depression, having received their first antidepressant prescription of the observation period within the first 3 months and were thus exempt from screening. The remaining 114,473 (91.5% of total sample size) patients were eligible for depression screening. However, depression screening was only undertaken in 35,537 (31.1% of those eligible) and 78,936 (68.9%) were not screened.

Table 1 provides the details of the demographic characteristics of those with known depression status and those with unknown depression status, along with 95% confidence intervals (CIs) for the differences between them. These show that, due to the extremely large sample size, there were significant differences between the two groups for all characteristics except smoking (for which there is a high level of missing data, likely to be biased since most of the data on non-smokers was missing). However the CIs show that the magnitude of all of the differences was extremely small, suggesting that those screened were similar to those not screened in terms of their demographics.

**Table 1:** Comparison of patients with known depression status versus those with unknown depression status.

		Depression Status Unknown (N=73947)	Depression Status Known (N=51196)	95% CI for the difference between known and unknown
**Age (years)**	Mean (SD)	67.1 (14.4)	67.7 (12.3)	(0.42, 0.72)
	Median (IQR)	69.0 (58, 78)	69.0 (60, 77	
	*missing*	*30*	*12*	
**Age (years)**	18-44	5265 (7.1%)	1951 (3.8%)	(-3.6%, -3.1%)
	45-64	23225 (31.4%)	17270 (33.7%)	(1.8%, 2.9%)
	65-75	19390 (26.2%)	15082 (29.5%)	(2.7%, 3.7%)
	76-90	26037 (35.2%)	16881 (33.0%)	(-2.8%, -1.7%)
	*missing*	*30*	*12*	
**Sex**	Female	33210 (44.9%)	24356 (47.6%)	(2.1%, 3.2%)
	Male	40693 (55.1%)	26814 (52.4%)	
	*missing*	*44*	*26*	
**Quintile (most deprived = 1)**	1	27708 (38.5%)	21065 (42.0%)	(3.0%, 4.1%)
	2	14335 (19.9%)	9567 (19.1%)	(-1.3%, -0.4%)
	3	11124 (15.5%)	6272 (12.5%)	(-3.3%, -2.5%)
	4	9056 (12.6%)	5422 (10.8%)	(-2.1%, -1.4%)
	5	9746 (13.5%)	7775 (15.5%)	(1.6%, 2.4%)
	*missing*	*1978*	*1095*	
**Co-morbidity**	Single diagnosis	61445 (83.1%)	39774 (77.7%)	(-5.9%, -5.0%)
	Two diagnoses	11355 (15.4%)	10311 (20.1%)	(4.3%, 5.2%)
	Three diagnoses	1147 (1.6%)	1111 (2.2%)	(0.5%, 0.8%)
	*missing*	*0*	*0*	
**Smoking**	Current non-smoker	14484 (61.3%)	13236 (61.8%)	(0.4%, 1.4%)
	Smoker	9144 (38.7%)	8178 (38.2%)	
	*missing*	*50319*	*29782*	
**Alcohol (units/week)**	Moderate	17689 (95.6%)	37030	(0.7%, 1.4%)
	Hazardous	673 (3.6%)	1138 (3.0%)	(-1.0%, -0.3%)
	Harmful	138 (0.8%)	149 (0.4%)	(-0.5%, -0.2%)
	*missing*	*55447*	*12879*	

*Mean (SD*)* and median (IQR*)* are presented for continuous variables and count (%*)* for categorical*.

### Section 2. Impact of Depression Screening on Antidepressant Prescribing

Figure 2 shows that, of the screened patients, 7080 (19.9%) were identified as screen positives based on HADS ≥8, of which 572 (8%) (relative risk against those with HADS<8, 3.3 (CI 2.97-3.67), p value <0.0001) were initiated on new antidepressant treatment within the observation period. Only 2925 (8.2% of screened) patients had an indication of moderate to severe depression with HADS ≥11 and of these 303 (10.4%) received a new anti-depressant prescription within 6 months of their assessment. This was the threshold recommended by the health board for considering pharmacotherapy. Finally, 696 (2.4%) of 28,457 patients with a normal HADS (<8) were also started on new antidepressants during the observation period.

Figure 2 also shows that 4989 patients in the non-screened population of 78,936 received their first antidepressant prescription in the last 9 months of observation period; these patients were ‘likely’ to be newly treated based on the assumption outlined in the methods section. Thus, the estimated overall new antidepressant prescription rate for the non-screened population was 6.3%. The overall new antidepressant prescription rate for the screened population was 3.5% (1268 patients out of 35,537). The new antidepressant prescription rate was higher for the non-screened population than for the screened population but it was lower than the rate for those with a raised HADS (≥8), which was 8%.

**Figure 3:**
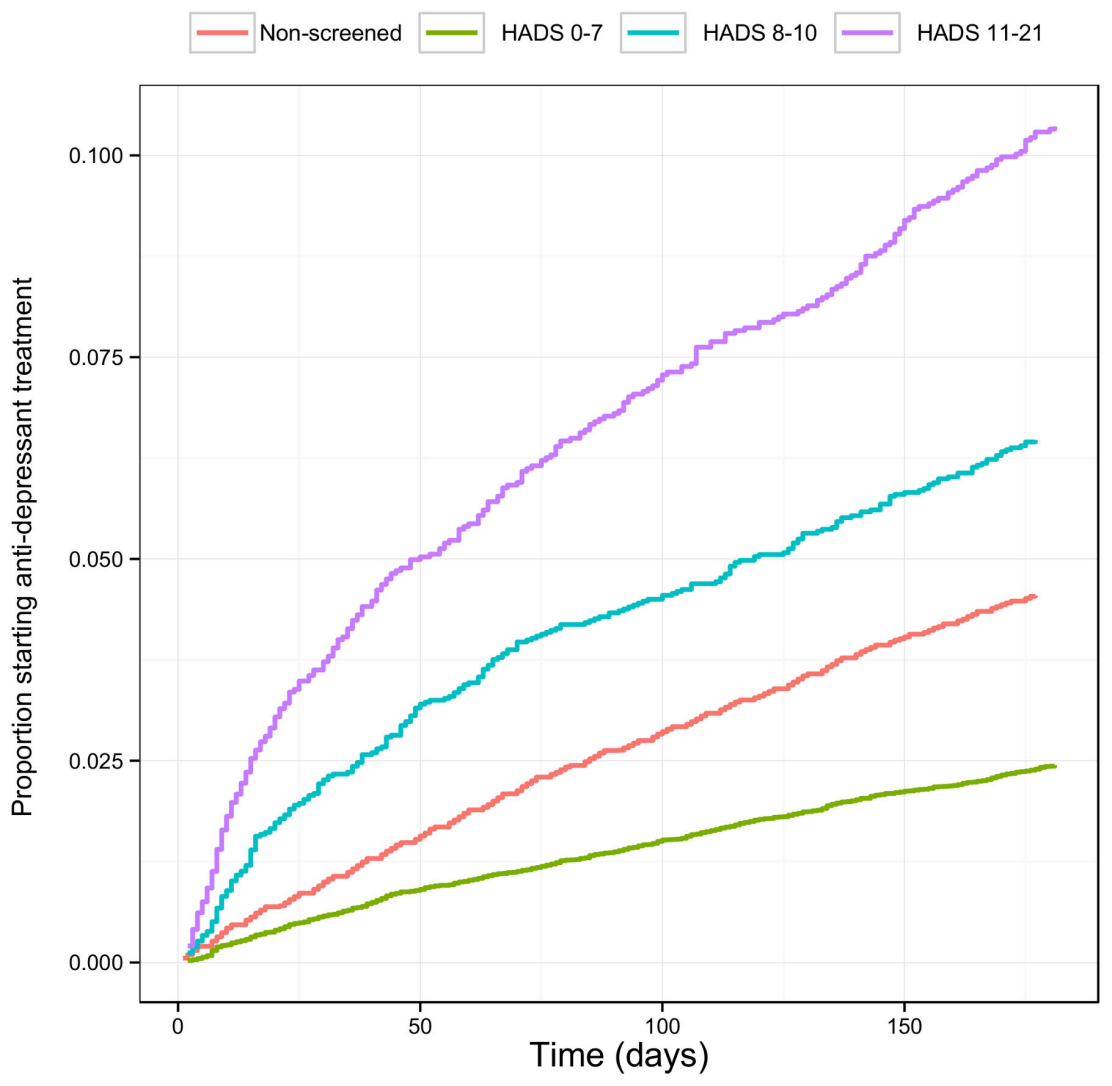
Antidepressant initiation time for the screened and non-screened population.

Figure 3 presents the time to starting new treatments for the screened and non-screened populations. This illustrates the association between a raised HADS (≥8) recorded at screening and a greater proportion of patients receiving treatment more quickly. It is also notable that subjects identified with normal range HADS (<8) receive the lowest rate of new treatments; even lower than the overall rate of the non-screened population.

As a sensitivity analysis, we undertook the analyses with and without amitriptyline, since it is often used for other indications, such as diabetic neuropathy. While there were lower proportions of patients recorded as being on antidepressants when amitriptyline was excluded, the distribution across the HADS categories was similar. Appendix S2 reports the full list of antidepressants prescribed with details of number of patients for the non-screened and the screened population.

### Section 3. Multimorbidity and Depression Screening

Table 2 compares the uptake of depression screening and the rate of positive depression screening (HADS ≥8) between patients with one of the three chronic diseases under study, those with any combination of two diseases and those with all three chronic diseases. The proportions for uptake and positive screening result increase along with increasing number of chronic conditions. Patients with multimorbidity were significantly more likely to have a positive result on screening (OR 1.48 (95% CI 1.39-1.58) (Table 3).

**Table 2:** Depression screening uptake and proportion of positive depression screening found across diagnostic subgroups.

	Single Diagnosis			Multiple Diagnoses	
	**Stroke (N=14447**)	**Diabetes (N=44247**)	**CHD (N=42525**)	Two Diseases* (N=21666)	**All Diseases (N=2258**)
Eligible for Screening	12485	37601	38715	18765	1918
Subjects Screened	3558 (28%)	12082 (32%)	11716 (30%)	7410 (39%)	771 (40%)
Positive Screening (HADS ≥8)	753 (21%)	2071 (17%)	2271 (19%)	1781 (24%)	204 (26%)

* Subject has two diagnoses of stroke, CHD or diabetes.

**Table 3:** Regression Tables.

**Odds Ratio of having raised HADS (≥8**)** for patients who underwent depression screening, adjusted for listed variables**			Odds Ratio of starting new antidepressants for patients with raised HADS (≥8), adjusted for listed variables		
	**Odds Ratio (95% CI**)	**p-value**		**Odds Ratio (95% CI**)	**p-value**
			**HADS (per unit increase**)	**1.11 (1.07, 1.14)**	**<0.001**
**Age (vs. 76 to 90)**			**Age (vs. 76 to 90**)		
**65 to 75**	**1.10 (1.02, 1.17)**	**0.010**	**65 to 75**	**1.56 (1.21, 2.02)**	**0.001**
**45 to 64**	**1.81 (1.69, 1.94)**	**˂0.001**	**45 to 64**	**2.00 (1.58,2.54**)	**˂0.001**
**18 to 44**	**1.76 (1.52, 2.05)**	**<0.001**	**18 to 44**	1.58 (0.97,2.57)	0.068
**Male (vs Female)**	**0.83 (0.79, 0.89)**	**<0.001**	**Male (vs Female**)	**0.63 (0.53, 0.75)**	**<0.001**
**Comorbidity (vs single disease)**	**1.48 (1.39, 1.58)**	**<0.001**	**Comorbidity (vs single disease**)	1.14 (0.94, 1.39)	0.200
**Deprivation Quintiles (vs. Q5-least deprived)**					
**Q4**	**1.27 (1.13, 1.43)**	**<0.001**	**Q4**	0.88 (0.58, 1.33)	0.541
**Q3**	**1.51 (1.35, 1.69**	**<0.001**	**Q3**	0.88 (0.59, 1.29)	0.507
**Q2**	**1.90 (1.72, 2.11)**	**<0.001**	**Q2**	0.74 (0.52, 1.05)	0.094
**Q1 - most deprived**	**2.56 (2.34, 2.79)**	**<0.001**	**Q1 - most deprived**	0.88 (0.65, 1.19)	0.402

### Section 4. Patient demographics, depression screening and antidepressant initiation

**Figure 4:**
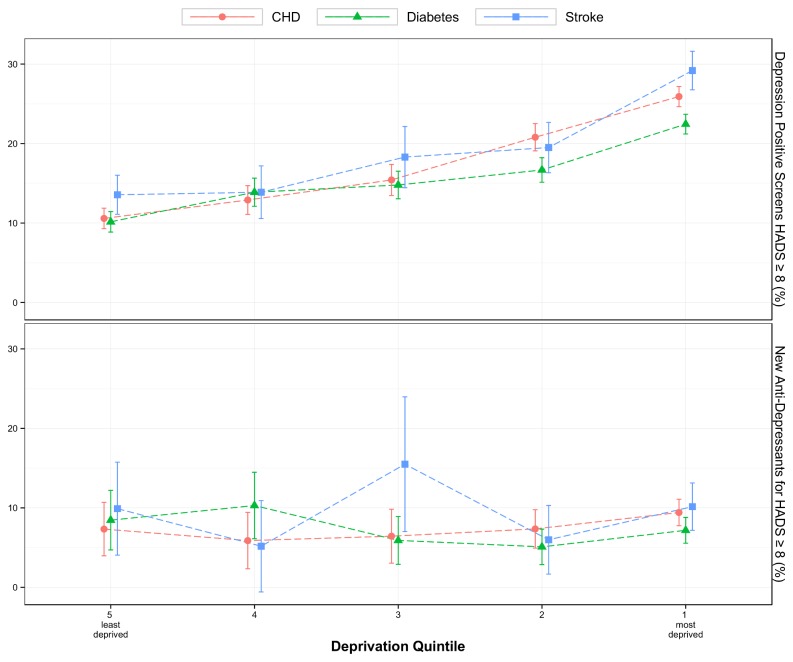
Title-Depression Screening and Socioeconomic deprivation. Rate of positive depression screening found from total screened (upper plot); and rate of anti-depressant initiation from those with raised HADS ≥8 (lower plot), versus socioeconomic deprivation status.

Figure 4 illustrates the clear socioeconomic gradient in the rate of having a positive depression screen (HADS ≥8), with rates being highest amongst those who were most deprived. Logistic regression results shown in Table 3 confirm that patients from the most deprived areas were more than twice as likely to have a positive result (HADS ≥8) as compared to those from the least deprived areas (OR 2.56, 95% CI 2.34-2.79). However, socioeconomic status did not have a significant impact on probability of starting a new antidepressant among those with a positive depression screen.

**Figure 5:**
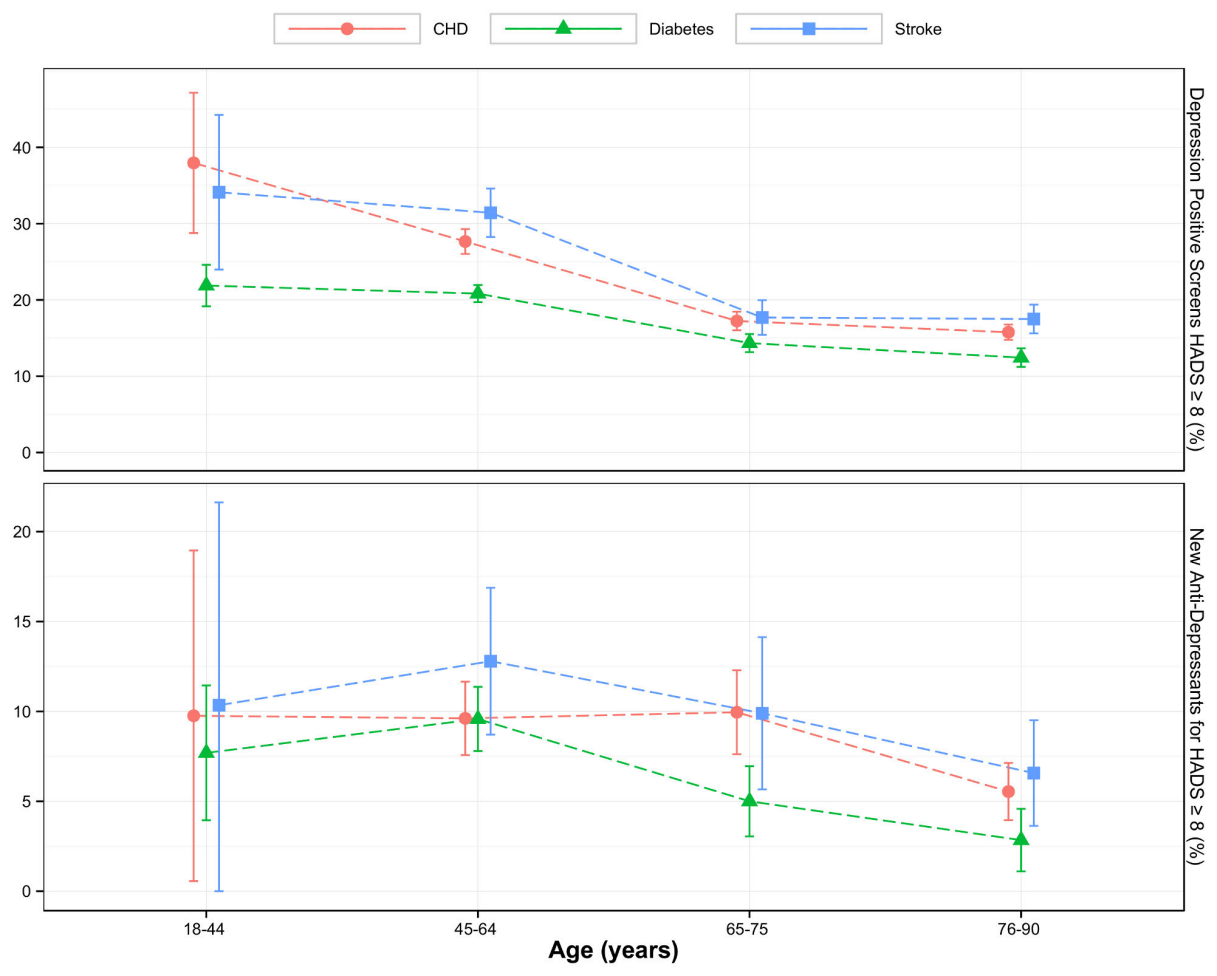
Title- Depression screening and Age. Rate of positive depression screening found from total screened (upper plot); and rate of antidepressants initiation from those with raised HADS ≥8 (lower plot) versus age.

Figure 5 shows that patients from older age groups were less likely to have a raised HADS (≥8) on depression screening as compared to their younger counterparts. The youngest and oldest patients appeared to have lower rates of initiation of antidepressants given a raised HADS (≥8) than those in middle age. Table 3 confirms that the patients aged 45-64 were at least 1.5 times more likely to be treated with antidepressants given a raised HADS than the oldest patients (OR 2.00, 95% CI 1.58-2.54).

Table 3 also shows that males were significantly less likely than females to have a positive result on depression screening (OR 0.83, 95%CI 0.79-0.89). Amongst positive depression screens with HADS ≥8, males were also at least 25% less likely than females to be initiated on antidepressants (OR 0.63, CI 0.53-0.75).

**Figure 6:**
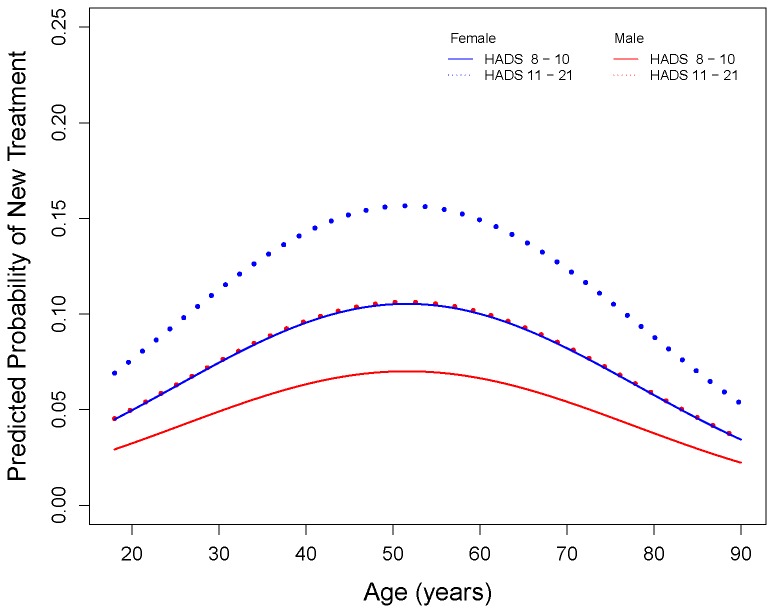
Relationship between probability of new antidepressant treatment, gender, and age and HADS categories, adjusted for deprivation and comorbidity.

An increase in HADS was associated with an increase in the rate of initiation of antidepressants, with each unit increase above 8 in HADS increasing the odds of treatment by 11% (OR 1.11, CI 1.07-1.14). Figure 6 displays the predicted probabilities of a subject starting new treatment given a raised HADS (≥8) against age for each gender and HADS category, showing how much more likely middle-aged and female patients were to start treatment than the oldest, youngest and male patients, for both categories of raised HADS.

## Discussion

### Summary of Findings and Comparison with Existing Literature

Despite the fact that primary care practitioners were being incentivized to screen for depression in chronic disease, a majority of patients (68.9%) were not documented as having been screened during the observation period. Depression screening was low in our study. While inaccuracies in recording the results on the health assessment template could account for some of the missing results for depression screening in the database, it seems unlikely that this could provide a full explanation of our findings. Potential explanations include lack of family practitioner confidence in the value of HADS-D, it has lower overall accuracy when compared to other depression screening tools [17]; or lack of family practitioner support for this type of screening due to lack of evidence that systematic depression screening leads to improvement in clinical outcomes [15,16,32]. Alternatively, previously reported barriers to discussing depression (or mental health) in patients with chronic disease in primary care, such as stigma associated around the ‘label’ and physicians’ preconception of normalizing depression in patients with chronic disease, could be influencing factors [33,34].

The rate of positive screens identified as a result of depression screening in our study of routine practice ranged from 17-21% for those with a single condition to 26% for those with multimorbidity. This is consistent with rates of 6-22% which have been reported in clinical trials of depression screening [15]. However, epidemiological studies have reported varying results. In a study of routine primary care involving more than 10,000 patients in the United States, a yield of 20.1% positive screens was reported using the PHQ-2 score [35]; while a similar study of 532 patients in Norfolk reported a much smaller yield of 2.3% using the two stem questions (‘During the last month, have you often been bothered by feeling down, depressed or hopeless?’ and ‘During the last month, have you often been bothered by having little interest or pleasure in doing things?’) as a screening tool [36]. Interestingly, Burton et al reported a much lower rate of 3% of new depression cases found from depression screening using the validated two stem questions [37], based on their analysis of a large population study from a different database [38]. However, it is difficult to make a direct comparison of our results with those of Burton et al as their caseness criteria for depression relied on diagnostic coding entered by health professionals, which in turn depends on how health professionals interpret and manage a positive result on depression screening and data on the rate of positive screens is unavailable for that study. HADS-D has a specificity of 81%, when used for depression screening in patients with chronic physical health problems in a primary care setting [17]. It is likely that some of the ‘positive screens’ identified in our study include false positive cases and hence we over-estimate the yield identified from depression screening. On the other hand, there is evidence to suggest that family practitioners often avoid using specific diagnostic codes while recording depression, which underestimates the actual occurrence in clinical practice [39,40].

There is growing evidence internationally, that multimorbidity is the norm rather than the exception, particularly in ageing populations [41-43]. Our study shows a clear relationship between the number of chronic diseases and number of positive screens found on depression screening. Similar associations between multimorbidity and depression screening have been reported from a study based on results from a self reported postal survey in Australian primary care [44]. This further strengthens the argument put forward by policy makers and researchers, both in the UK and US, for the need to consider physical and mental health needs together [45-47].

Importantly, we have shown that higher HADS on depression screening was associated with higher rates of new antidepressant prescriptions in routine primary care practice. This is contrary to previous evidence which has suggested that depression screening does not lead to increases in antidepressant prescribing [13,32]. However, our study reports what happens in routine clinical practice, whereas the existing evidence draws on findings from randomized controlled trials with small sample sizes and studies that usually did not focus on screening for depression comorbid with chronic disease. The majority of those prescribed antidepressants within six months of depression screening had either a negative screening result (HADS <8) or a positive screening with a probability of mild depression (HADS 8-10). There is little evidence of additional benefit of antidepressants compared with placebo in this group of patients [28] and hence this finding has important clinical and resource implications. However, this should be considered in the context of recent evidence of rising antidepressant prescribing in Scotland and the UK, and the uncertainty whether this rise should be attributed to increase in case-identification of depression or increase in duration of depression treatment or both [48-50]. The new antidepressant prescribing rate in the non-screened population was lower than those with raised HADS but higher than those with a normal HADS.

Various studies have shown that females and patients who are from socioeconomically deprived backgrounds are more likely to develop depression [51,52] but this study is one of the first to study their impact on results of depression screening in chronic disease. Our study showed that among the new depression cases, there was no difference in the rate of antidepressant initiation amongst different socioeconomic groups. However, we did find that males were less likely to be prescribed antidepressants, to the extent that a man with HADS ≥11 had a similar chance of being prescribed antidepressants as a woman with HADS of 8-10. This is contrary to the previous evidence which shows that family practitioners were more likely to prescribe antidepressants to male patients presenting with depression [53,54] suggesting there needs to be further research in this area to study the impact of gender on depression treatment. We also showed that middle-aged patients were more likely than the oldest patients to be initiated on antidepressants, given a raised HADS. Previous evidence has shown that primary care professionals often have ‘therapeutic nihilism’ when dealing with depression in elderly patients [55], which could be one of the explanations for this observed difference.

### Strengths

This is the first study, to our knowledge, to examine the impact of implementing systematic depression screening for patients with chronic disease in routine clinical practice. The study has a large sample size with good representation of patients from different age groups and socioeconomic backgrounds. Processing of the large and complex dataset was cross validated by two researchers independently for quality control and robustness. A standardized and validated method of depression screening was used.

### Limitations

This study used routinely collected data that was not initially intended to be used for research purposes. Since only a minority of the patients were actually screened, depression status was unknown for a large number of patients; also information on health related behaviour was incomplete in the majority of patients. There may be important differences between patients with known depression status and those whose depression status was unknown, which are not evident from their baseline demographic data. Practitioners may intuitively screen those patients where they are more likely to get a positive result, for instance patients with multimorbidity. Also, there is a possibility of reverse causality with GPs reviewing a patient whom they consider to have depression and offering screening subsequently. The overall accuracy of depression screening in our study was reliant on HADS-D which may not be the best available screening tool for patients with chronic disease in a primary care setting [17]. The uptake for depression screening was much lower in our study when compared with uptake for depression screening in the QOF programme in Scotland (90.5%) for the year 2008-09 [56]. However, the QOF programme had target driven incentivisation where practices were only paid if they achieved 90% coverage in depression screening. The incentivisation for the LES programme used in our study instead depended on achieving a proportion of clinical indicators across the health assessment protocol. Secondly the screening tool used for the QOF programme is two stem questions which are much quicker to administer when compared to the HADS-D questionnaire which consists of 7 items. Finally we did not have information on the presence of chronic conditions outside the ones studied, thus multimorbidity count is likely to be underestimated, nor on the number of patients referred for psychological therapies following their depression screening and assessment. We also did not have information on previous episodes of depression or previous treatment with antidepressants prior to the observation period, which are likely to be important influencing factors on antidepressant initiation rate.

### Implications for Practice

Implementation of systematic depression screening for patients with chronic disease needs careful consideration. Our study shows that even within the context of incentivisation, universal coverage can be difficult to achieve in routine practice, unless simpler screening tools such as two stem questions are used. Importantly, depression screening identified large numbers of new cases which has clear resource implications, especially since it was clear that depression screening was associated with increases in anti-depressant prescribing and antidepressants were prescribed to patients where there is little evidence of additional benefit above placebo (HADS<8 and HADS 8-10).

Our data suggests that younger individuals, those with multimorbidity, and those from more deprived backgrounds were more at risk of depression in this population, thus suggesting that targeting depression screening at high risk groups could be an alternative approach. Males and elderly patients were less likely to be initiated on antidepressants, which needs further investigation.

## Conclusion

The reach of depression screening in routine practice was low in our study despite incentivisation. A simpler screening tool or targeting high risk patient groups may be more feasible in routine practice, but needs further research. In our study, identification of depression in chronic disease was associated with treatment changes in routine practice. The key question will be to explore how such treatment changes affect outcomes, if at all.

## Supporting Information

Appendix S1 (XLS)

Appendix S2 (DOCX)

## References

[1] Egede LE (2007) Major depression in individuals with chronic medical disorders: prevalence, correlates and association with health resource utilization, lost productivity and functional disability. Gen Hosp Psychiatry 29: 409-416. doi:10.1016/j.genhosppsych.2007.06.002. PubMed: 17888807. 17888807 10.1016/j.genhosppsych.2007.06.002

[2] Moussavi S, Chatterji S, Verdes E, Tandon A, Patel V, Ustun B (2007) Depression, chronic diseases, and decrements in health: results from the World Health Surveys. Lancet 370: 851-858. doi:10.1016/S0140-6736(07)61415-9. PubMed: 17826170. 17826170 10.1016/S0140-6736(07)61415-9

[3] Mitchell AJ, Ferguson DW, Gill J, Paul J, Symonds P (2013) Depression and anxiety in long-term cancer survivors compared with spouses and healthy controls: a systematic review and meta-analysis. Lancet Oncol 14: 721-732. S1470- doi:10.1016/S1470-2045(13)70244-4. PubMed: 23759376. 2045(13)70244-4 . PII . doi:10.1016/S1470-2045(13)70244-4 23759376 10.1016/S1470-2045(13)70244-4

[4] Ali S, Stone MA, Peters JL, Davies MJ, Khunti K (2006) The prevalence of co-morbid depression in adults with Type 2 diabetes: a systematic review and meta-analysis. Diabet Med 23: 1165-1173. doi:10.1111/j.1464-5491.2006.01943.x. PubMed: 17054590. 17054590 10.1111/j.1464-5491.2006.01943.x

[5] Hadidi N, Treat-Jacobson DJ, Lindquist R (2009) Poststroke depression and functional outcome: a critical review of literature. Heart Lung 38: 151-162. doi:10.1016/j.hrtlng.2008.05.002. PubMed: 19254633. 19254633 10.1016/j.hrtlng.2008.05.002

[6] Whooley MA, de Jonge P, Vittinghoff E, Otte C, Moos R et al. (2008) Depressive symptoms, health behaviors, and risk of cardiovascular events in patients with coronary heart disease. JAMA 300: 2379-2388. doi:10.1001/jama.2008.711. PubMed: 19033588. 19033588 10.1001/jama.2008.711 PMC2677371

[7] Katon WJ, Rutter C, Simon G, Lin EH, Ludman E et al. (2005) The association of comorbid depression with mortality in patients with type 2 diabetes. Diabetes Care 28: 2668-2672. doi:10.2337/diacare.28.11.2668. PubMed: 16249537. 16249537 10.2337/diacare.28.11.2668

[8] Pozuelo L, Tesar G, Zhang J, Penn M, Franco K et al. (2009) Depression and heart disease: what do we know, and where are we headed? Cleve Clin J Med 76: 59-70. doi:10.3949/ccjm.75a.08011. PubMed: 19122112. 19122112 10.3949/ccjm.75a.08011

[9] International Diabetes Federation (2005) IDF Clinical Guidelines Task Force Global Guidelines for Type 2 Diabetes. Available: https://www.idf.org/webdata/docs/IDF%20GGT2D.pdf. Accessed 18^th^ February 2013.

[10] Lichtman JH, Bigger JT Jr., Blumenthal JA, Frasure-Smith N, Kaufmann PG et al. (2008) Depression and coronary heart disease: recommendations for screening, referral, and treatment: a science advisory from the American Heart Association Prevention Committee of the Council on Cardiovascular Nursing, Council on Clinical Cardiology, Council on Epidemiology and Prevention, and Interdisciplinary Council on Quality of Care and Outcomes Research: endorsed by the American Psychiatric Association. Circulation 118: 1768-1775. doi:10.1161/CIRCULATIONAHA.108.190769. PubMed: 18824640. 18824640 10.1161/CIRCULATIONAHA.108.190769

[11] Doran T, Fullwood C, Gravelle H, Reeves D, Kontopantelis E et al. (2006) Pay-for-performance programs in family practices in the United Kingdom. N Engl J Med 355: 375-384. doi:10.1056/NEJMsa055505. PubMed: 16870916. 16870916 10.1056/NEJMsa055505

[12] Goodyear-Smith FA, van Driel ML, Arroll B, Del MC (2012) Analysis of decisions made in meta-analyses of depression screening and the risk of confirmation bias: a case study. BMC Med Res Methodol 12: 76. doi:10.1186/1471-2288-12-76. PubMed: 22691262. 22691262 10.1186/1471-2288-12-76 PMC3464667

[13] Gilbody S, Sheldon T, House A (2008) Screening and case-finding instruments for depression: a meta-analysis. CMAJ 178: 997-1003. doi:10.1503/cmaj.070281. PubMed: 18390942. 18390942 10.1503/cmaj.070281 PMC2276549

[14] O’Connor EA, Whitlock EP, Beil TL, Gaynes BN (2009) Screening for depression in adult patients in primary care settings: a systematic evidence review. Ann Intern Med 151: 793-803. doi:10.7326/0003-4819-151-11-200912010-00007. PubMed: 19949145. 19949145 10.7326/0003-4819-151-11-200912010-00007

[15] Thombs BD, de Jonge P, Coyne JC, Whooley MA, Frasure-Smith N et al. (2008) Depression screening and patient outcomes in cardiovascular care: a systematic review. JAMA 300: 2161-2171. doi:10.1001/jama.2008.667. PubMed: 19001627. 19001627 10.1001/jama.2008.667

[16] Thombs BD, Roseman M, Coyne JC, de Jonge P, Delisle VC et al. (2013) Does evidence support the American Heart Association’s recommendation to screen patients for depression in cardiovascular care? An updated systematic review. PLOS ONE 8: e52654. doi:10.1371/journal.pone.0052654. PubMed: 23308116. PONE-D-12-27966 . PII. 23308116 10.1371/journal.pone.0052654 PMC3538724

[17] Meader N, Mitchell AJ, Chew-Graham C, Goldberg D, Rizzo M et al. (2011) Case identification of depression in patients with chronic physical health problems: a diagnostic accuracy meta-analysis of 113 studies. Br J Gen Pract 61: e808-e820. doi:10.3399/bjgp11X613151. PubMed: 22137418. 22137418 10.3399/bjgp11X613151 PMC3223779

[18] National Institute of Health and Clinical Excellence (2009) Depression in people with a chronic physical health problem. CG91. Available: http://www.nice.org.uk/guidance/index.jsp?action=download&o=45914 . Accessed; 18^th^ (Feb 2013).

[19] Thombs BD, Arthurs E, El-Baalbaki G, Meijer A, Ziegelstein RC et al. (2011) Risk of bias from inclusion of patients who already have diagnosis of or are undergoing treatment for depression in diagnostic accuracy studies of screening tools for depression: systematic review. BMJ 343: d4825. doi:10.1136/bmj.d4825. PubMed: 21852353. 21852353 10.1136/bmj.d4825 PMC3191850

[20] Roland M (2004) Linking physicians’ pay to the quality of care--a major experiment in the United Kingdom. N Engl J Med 351: 1448-1454. doi:10.1056/NEJMhpr041294. PubMed: 15459308. 15459308 10.1056/NEJMhpr041294

[21] Department of Health UK (2012) Read Codes. Available: http://www.connectingforhealth.nhs.uk/systemsandservices/data/uktc/readcodes. Accessed 18^th^ February 2013.

[22] Zigmond AS, Snaith RP (1983) The hospital anxiety and depression scale. Acta Psychiatr Scand 67: 361-370. doi:10.1111/j.1600-0447.1983.tb09716.x. PubMed: 6880820. 6880820 10.1111/j.1600-0447.1983.tb09716.x

[23] Living Life to the Full Course (2012) Living Life to the; Full. Available: http://www.llttf.com/index.php. Accessed 18^th^ February 2013.

[24] The STEPS team (2012) The. Glasgow: Steps. Available: http://glasgowsteps.com/about/serviceInformation.php . Accessed 18^th^ February 2013

[25] Bjelland I, Dahl AA, Haug TT, Neckelmann D (2002) The validity of the Hospital Anxiety and Depression Scale. An updated literature review. J Psychosom Res 52: 69-77. doi:10.1016/S0022-3999(01)00296-3. PubMed: 11832252. S0022399901002963 . PII. 11832252 10.1016/s0022-3999(01)00296-3

[26] Stafford L, Berk M, Jackson HJ (2007) Validity of the Hospital Anxiety and Depression Scale and Patient Health Questionnaire-9 to screen for depression in patients with coronary artery disease. Gen Hosp Psychiatry 29: 417-424. doi:10.1016/j.genhosppsych.2007.06.005. PubMed: 17888808. 17888808 10.1016/j.genhosppsych.2007.06.005

[27] Hansson M, Chotai J, Nordstöm A, Bodlund O (2009) Comparison of two self-rating scales to detect depression: HADS and PHQ-9. Br J Gen Pract 59: e283-e288. doi:10.3399/bjgp09X454070. PubMed: 19761655. 19761655 10.3399/bjgp09X454070 PMC2734374

[28] National Institute of Health and Clinical Excellence (2009) Depression in Adults. (update) CG 90 Available: http://publications.nice.org.uk/depression-in-adults-cg90 . Accessed; 18^th^ (Feb 2013).

[29] Information Services Division Scotland (2012) Scottish Index of Multiple Deprivation (SIMD). Available: http://www.scotland.gov.uk/Topics/Statistics/SIMD. Accessed 18^th^ February 2013.

[30] National Statistics Publication for Scotland (2013) Scottish Health Survey; (2009) Volume 1: Main Report. Available: http://www.scotland.gov.uk/Publications/2010/09/23154223/0. Accessed 17^th^ April 2013.

[31] The R Project for Statistical Computing R Core Team; (2012) R: A language and environment for statistical computing. Vienna, Austria: R Foundation for Statistical Computing ISBN 3-900051-07-0 Available: http://www.R-project.org. Accessed 18^th^ February 2013.

[32] Pouwer F, Tack CJ, Geelhoed-Duijvestijn PH, Bazelmans E, Beekman AT et al. (2011) Limited effect of screening for depression with written feedback in outpatients with diabetes mellitus: a randomised controlled trial. Diabetologia 54: 741-748. doi:10.1007/s00125-010-2033-0. PubMed: 21221528. 21221528 10.1007/s00125-010-2033-0 PMC3052512

[33] Coventry PA, Hays R, Dickens C, Bundy C, Garrett C et al. (2011) Talking about depression: a qualitative study of barriers to managing depression in people with long term conditions in primary care. BMC Fam Pract 12: 10. doi:10.1186/1471-2296-12-10. PubMed: 21426542. 21426542 10.1186/1471-2296-12-10 PMC3070666

[34] Karasz A, Dowrick C, Byng R, Buszewicz M, Ferri L et al. (2012) What we talk about when we talk about depression: doctor-patient conversations and treatment decision outcomes. Br J Gen Pract 62: e55-e63. doi:10.3399/bjgp12X616373. PubMed: 22520683. 22520683 10.3399/bjgp12X616373 PMC3252540

[35] Yano EM, Chaney EF, Campbell DG, Klap R, Simon BF et al. (2012) Yield of practice-based depression screening in VA primary care settings. J Gen Intern Med 27: 331-338. doi:10.1007/s11606-011-1904-5. PubMed: 21975821. 21975821 10.1007/s11606-011-1904-5 PMC3286554

[36] Croxford AM (2013) An Evaluation of Routine Screening, Assessment and Treatment of Depression for Patients on the Diabetes and / or Coronary Heart Disease Registers in a Primary Care Practice in Norfolk. Reinvention: an International Journal of Undergraduate Research. Available: http://www2.warwick.ac.uk/fac/cross_fac/iatl/ejournal/issues/volume3issue1/croxford/ . Accessed 17^th^ April 2013

[37] Whooley MA, Avins AL, Miranda J, Browner WS (1997) Case-finding instruments for depression. Two questions are as good as many 2. J Gen Intern Med 12: 439-445. doi:10.1046/j.1525-1497.1997.00076.x. PubMed: 9229283. 9229283 10.1046/j.1525-1497.1997.00076.x PMC1497134

[38] Burton C, Simpson C, Anderson N (2012) Diagnosis and treatment of depression following routine screening in patients with coronary heart disease or diabetes: a database cohort study. Psychol Med: 1-9. 10.1017/S0033291712001481 22804849

[39] Rait G, Walters K, Griffin M, Buszewicz M, Petersen I et al. (2009) Recent trends in the incidence of recorded depression in primary care. Br J Psychiatry 195: 520-524. doi:10.1192/bjp.bp.108.058636. PubMed: 19949202. 19949202 10.1192/bjp.bp.108.058636

[40] Joling KJ, van Marwijk HW, Piek E, van der Horst HE, Penninx BW et al. (2011) Do GPs’ medical records demonstrate a good recognition of depression? A new perspective on case extraction. J Affect Disord 133: 522-527. doi:10.1016/j.jad.2011.05.001. PubMed: 21605910. 21605910 10.1016/j.jad.2011.05.001

[41] Barnett K, Mercer SW, Norbury M, Watt G, Wyke S et al. (2012) Epidemiology of multimorbidity and implications for health care, research, and medical education: a cross-sectional study. Lancet 380: 37-43. doi:10.1016/S0140-6736(13)60393-1. PubMed: 22579043. 22579043 10.1016/S0140-6736(12)60240-2

[42] Fortin M, Bravo G, Hudon C, Vanasse A, Lapointe L (2005) Prevalence of multimorbidity among adults seen in family practice. Ann Fam Med 3: 223-228. doi:10.1370/afm.272. PubMed: 15928225. 15928225 10.1370/afm.272 PMC1466875

[43] Parekh AK, Barton MB (2010) The challenge of multiple comorbidity for the US health care system. JAMA 303: 1303-1304. doi:10.1001/jama.2010.381. PubMed: 20371790. 20371790 10.1001/jama.2010.381

[44] Gunn JM, Ayton DR, Densley K, Pallant JF, Chondros P et al. (2012) The association between chronic illness, multimorbidity and depressive symptoms in an Australian primary care cohort. Soc Psychiatry Psychiatr Epidemiol 47: 175-184. doi:10.1007/s00127-010-0330-z. PubMed: 21184214. 21184214 10.1007/s00127-010-0330-z

[45] Naylor C, Parsonage M, McDaid D, Knapp M, Fossey M et al. (2012) Long-term conditions and mental health.The cost of co-morbidities. King’s Fund and Centre for Mental Health. Available: http://www.kingsfund.org.uk/publications/long-term-conditions-and-mental-health. Accessed 18^th^ February 2013.

[46] US Department of Health and Human Services (2010) US Department of Health and Human Services. Multiple chronic conditions- a strategic framework: optimum health and quality of life for individuals with multiple chronic conditions. Available: http://www.hhs.gov/ash/initiatives/mcc/mcc_framework.pdf. Accessed 18^th^ February 2013.

[47] Mercer SW, Gunn J, Bower P, Wyke S, Guthrie B (2012) Managing patients with mental and physical multimorbidity. BMJ 345: e5559. doi:10.1136/bmj.e5559. PubMed: 22945951. 22945951 10.1136/bmj.e5559

[48] Munoz-Arroyo R, Sutton M, Morrison J (2006) Exploring potential explanations for the increase in antidepressant prescribing in Scotland using secondary analyses of routine data 2. Br J Gen Pract 56: 423-428. PubMed: 16762123. 16762123 PMC1839016

[49] Moore M, Yuen HM, Dunn N, Mullee MA, Maskell J et al. (2009) Explaining the rise in antidepressant prescribing: a descriptive study using the general practice research database 2. BMJ 339: b3999. doi:10.1136/bmj.b3999. PubMed: 19833707. 19833707 10.1136/bmj.b3999 PMC2762496

[50] Lockhart P, Guthrie B (2011) Trends in primary care antidepressant prescribing 1995-2007: a longitudinal population database analysis 1. Br J Gen Pract 61: e565-e572. doi:10.3399/bjgp11X593848. PubMed: 22152736. 22152736 10.3399/bjgp11X593848 PMC3162179

[51] Lorant V, Deliège D, Eaton W, Robert A, Philippot P et al. (2003) Socioeconomic inequalities in depression: a meta-analysis. Am J Epidemiol 157: 98-112. doi:10.1093/aje/kwf182. PubMed: 12522017. 12522017 10.1093/aje/kwf182

[52] Scheibe S, Preuschhof C, Cristi C, Bagby RM (2003) Are there gender differences in major depression and its response to antidepressants? J Affect Disord 75: 223-235. doi:10.1016/S0165-0327(02)00050-2. PubMed: 12880935. 12880935 10.1016/s0165-0327(02)00050-2

[53] Dumesnil H, Cortaredona S, Verdoux H, Sebbah R, Paraponaris A et al. (2012) General practitioners’ choices and their determinants when starting treatment for major depression: a cross sectional, randomized case-vignette survey. PLOS ONE 7: e52429. doi:10.1371/journal.pone.0052429. PubMed: 23272243. 23272243 10.1371/journal.pone.0052429 PMC3525552

[54] Hyde J, Evans J, Sharp D, Croudace T, Harrison G et al. (2005) Deciding who gets treatment for depression and anxiety: a study of consecutive GP attenders. Br J Gen Pract 55: 846-853. PubMed: 16282000. 16282000 PMC1570785

[55] Burroughs H, Lovell K, Morley M, Baldwin R, Burns A et al. (2006) 'Justifiable depression': how primary care professionals and patients view late-life depression? A qualitative Study Fam Pract 23: 369-377 10.1093/fampra/cmi115 16476699

[56] Information Services Division S (2013) General Practice- quality and outcomes framework, 2008/9 achievement summaries at practice level. Available: http://www.isdscotlandarchive.scot.nhs.uk/isd/6005.html. Accessed 17^th^ April 2013.

