# Supplementary material for: Challenges and Implications of Routine Depression Screening for Depression in Chronic Disease and Multimorbidity: A Cross Sectional Study
Source: PLoS One. 2013 Sep 13;8(9):e74610. doi: 10.1371/journal.pone.0074610 (PMC3772931; doi:10.1371/journal.pone.0074610)
Supplement: Appendix S2 — (DOCX) [file pone.0074610.s002.docx]

| **Appendix 2: Number (%) of individual drug scripts. Any anti-depressant prescribed for the non-screened population in 08/09 is listed Any anti-depressant prescribed for the screened population within 180 days post-screening is listed. Some subjects were prescribed combinations of drugs.** | | | | |
| --- | --- | --- | --- | --- |
| **Drugs** | **Non-screened** | **HADS 0-7** | **HADS 8-10** | **HADS 11-21** |
| Amitriptyline | 9 (0%) | 0 (0%) | 0 (0%) | 0 (0%) |
| Amitriptyline Hydrochloride | 4593 (24.1%) | 325 (43.4%) | 82 (27.1%) | 56 (16.4%) |
| Anafranil | 3 (0%) | 0 (0%) | 0 (0%) | 0 (0%) |
| Anafranil Sr | 7 (0%) | 0 (0%) | 0 (0%) | 0 (0%) |
| Cipramil | 2 (0%) | 0 (0%) | 0 (0%) | 0 (0%) |
| Citalopram | 4247 (22.3%) | 171 (22.9%) | 83 (27.4%) | 105 (30.7%) |
| Citalopram Hydrobromide | 897 (4.7%) | 17 (2.3%) | 7 (2.3%) | 6 (1.8%) |
| Citalopram Hydrochloride | 4 (0%) | 0 (0%) | 0 (0%) | 0 (0%) |
| Clomipramine Hydrochloride | 96 (0.5%) | 0 (0%) | 0 (0%) | 1 (0.3%) |
| Clomipramine Hydrochloride Mr | 4 (0%) | 0 (0%) | 0 (0%) | 0 (0%) |
| Depixol | 3 (0%) | 0 (0%) | 0 (0%) | 0 (0%) |
| Dosulepin | 145 (0.8%) | 3 (0.4%) | 1 (0.3%) | 1 (0.3%) |
| Dosulepin Hydrochloride | 421 (2.2%) | 6 (0.8%) | 5 (1.7%) | 4 (1.2%) |
| Doxepin | 22 (0.1%) | 1 (0.1%) | 0 (0%) | 0 (0%) |
| Efexor | 1 (0%) | 0 (0%) | 0 (0%) | 0 (0%) |
| Efexor Xl | 88 (0.5%) | 0 (0%) | 1 (0.3%) | 1 (0.3%) |
| Escitalopram | 57 (0.3%) | 0 (0%) | 0 (0%) | 0 (0%) |
| Fluoxetine | 2937 (15.4%) | 93 (12.4%) | 62 (20.5%) | 76 (22.2%) |
| Fluoxetine Hydrochloride | 597 (3.1%) | 8 (1.1%) | 6 (2%) | 8 (2.3%) |
| Flupentixol | 56 (0.3%) | 3 (0.4%) | 2 (0.7%) | 4 (1.2%) |
| Flupentixol Decanoate | 5 (0%) | 0 (0%) | 0 (0%) | 0 (0%) |
| Fluvoxamine Maleate | 4 (0%) | 0 (0%) | 0 (0%) | 0 (0%) |
| Imipramine | 41 (0.2%) | 2 (0.3%) | 0 (0%) | 1 (0.3%) |
| Imipramine Hydrochloride | 148 (0.8%) | 9 (1.2%) | 3 (1%) | 3 (0.9%) |
| Isocarboxazid | 2 (0%) | 0 (0%) | 0 (0%) | 0 (0%) |
| Lofepramine | 299 (1.6%) | 9 (1.2%) | 5 (1.7%) | 5 (1.5%) |
| Lustral | 4 (0%) | 0 (0%) | 0 (0%) | 0 (0%) |
| Manerix | 1 (0%) | 0 (0%) | 0 (0%) | 0 (0%) |
| Mianserin Hydrochloride | 7 (0%) | 0 (0%) | 0 (0%) | 0 (0%) |
| Mirtazapine | 524 (2.8%) | 9 (1.2%) | 6 (2%) | 15 (4.4%) |
| Moclobemide | 13 (0.1%) | 0 (0%) | 1 (0.3%) | 0 (0%) |
| Molipaxin | 1 (0%) | 0 (0%) | 0 (0%) | 0 (0%) |
| Nortriptyline | 97 (0.5%) | 8 (1.1%) | 2 (0.7%) | 1 (0.3%) |
| Optimax | 1 (0%) | 0 (0%) | 0 (0%) | 0 (0%) |
| Paroxetine | 645 (3.4%) | 9 (1.2%) | 9 (3%) | 3 (0.9%) |
| Paroxetine Hydrochloride | 288 (1.5%) | 4 (0.5%) | 4 (1.3%) | 1 (0.3%) |
| Phenelzine | 17 (0.1%) | 0 (0%) | 0 (0%) | 0 (0%) |
| Prothiaden | 7 (0%) | 0 (0%) | 0 (0%) | 0 (0%) |
| Prozac | 8 (0%) | 0 (0%) | 0 (0%) | 0 (0%) |
| Reboxetine | 51 (0.3%) | 0 (0%) | 0 (0%) | 1 (0.3%) |
| Seroxat | 8 (0%) | 0 (0%) | 0 (0%) | 0 (0%) |
| Sertraline | 714 (3.7%) | 13 (1.7%) | 5 (1.7%) | 10 (2.9%) |
| Sertraline Hydrochloride | 232 (1.2%) | 4 (0.5%) | 3 (1%) | 5 (1.5%) |
| Sinepin | 1 (0%) | 0 (0%) | 0 (0%) | 0 (0%) |
| Surmontil | 2 (0%) | 0 (0%) | 0 (0%) | 0 (0%) |
| Tranylcypromine | 4 (0%) | 0 (0%) | 0 (0%) | 0 (0%) |
| Trazodone Hydrochloride | 1045 (5.5%) | 41 (5.5%) | 13 (4.3%) | 28 (8.2%) |
| Trimipramine | 11 (0.1%) | 0 (0%) | 0 (0%) | 0 (0%) |
| Trimipramine Maleate | 12 (0.1%) | 0 (0%) | 0 (0%) | 0 (0%) |
| Tryptophan | 1 (0%) | 0 (0%) | 0 (0%) | 0 (0%) |
| Venlafaxine | 327 (1.7%) | 8 (1.1%) | 1 (0.3%) | 2 (0.6%) |
| Venlafaxine Mr | 332 (1.7%) | 5 (0.7%) | 2 (0.7%) | 5 (1.5%) |
| Zispin Soltab | 2 (0%) | 0 (0%) | 0 (0%) | 0 (0%) |
